# Paternal weight of ducks may have an influence on offspring’ small intestinal function and cecal microorganisms

**DOI:** 10.1186/s12866-020-01828-1

**Published:** 2020-06-05

**Authors:** Mingxia Ran, Bo Hu, Lumin Cheng, Shenqiang Hu, Hehe Liu, Liang Li, Jiwei Hu, Jiwen Wang

**Affiliations:** Farm Animal Genetic Resources Exploration and Innovation Key Laboratory of Sichuan Province, Sichuan Agricultural University, Chengdu, 611130 China

**Keywords:** Duck, Intestinal morphology, Digestive enzyme, Microbiota composition

## Abstract

**Background:**

In animals, many factors affect the small intestinal function and cecal microorganisms, including body weight and genetic background. However, whether paternal weight impacts the small intestinal function and cecal microorganisms remains unknown to date. The current study used Nonghua sheldrake to estimate the effect of paternal weight on the intestine of the offspring by evaluating differences in small intestinal morphology, digestive enzyme activity, and cecal microorganisms between the offspring of male parents with high body weight (group H) and that of male parents with low body weight (group L).

**Results:**

The results of the analysis of small intestinal morphology showed that the villus height of the jejunum of group H ducks was higher than that of group L ducks, and the difference was significant for ducks at 10 weeks of age. Moreover, the villus height/crypt depth of the duodenum in group H significantly exceeded that of group L at a duck age of 2 weeks. The amylase activity in the jejunum content of group H exceeded that of group L at 5 and 10 weeks of age. Furthermore, the proportion of the *Firmicutes* to *Bacteroidetes* was significantly higher in group H (duck age of 2 weeks). Among the genera with a relative abundance exceeding 1%, the relative abundances of genera *Desulfovibrio*, *Megamonas*, *Alistipes*, *Faecalibacterium*, and *Streptococcus* observed in group H were significantly different between group H and group L.

**Conclusions:**

For the first time, this study identifies the effect of paternal weight on offspring small intestinal function and cecal microorganisms. Consequently, this lays a foundation for further research on the relationship between male parents and offspring intestinal function.

## Background

The intestine is not only a vital organ responsible for the absorption of nutrients and the excretion of bile and waste but also a major site of host immunity [1]. In the poultry industry, appropriate intestinal function is of great importance to achieve target growth rates and feed efficiency [2]. It has been shown that dietary threonine (Thr) affects the Bifidobacterium abundance in laying hens and thus improves their gut function and further increases their laying performance [3]. Moreover, Thr availability is closely related to the function of gut barriers, which further contributes to body weight gain [4]. In addition to its effect on production performance, the intestinal mucosal barrier is also the first line of defence against hostile luminal environments, and it is therefore central for organic health [3].

Many factors indirectly influence intestinal function by impacting intestinal morphology, digestive enzyme activity, and the microbiome [5, 6]. Among these factors, bodyweight is particularly critical and should therefore not be neglected. In macroscopic anatomy, the gut differs relative to bodyweight because of the influence it exerts on intestinal morphology. For instance, in newborn rabbits, accelerated weight gain after birth induces premature maturity of the small intestinal epithelium [7]. Moreover, after divergent selection for bodyweight, mice showed significantly different villus height dependent on bodyweight [8]. Previous reports also highlighted that bodyweight is involved in the regulation of digestive enzyme activities. For example, 3 days post hatching, the levels of trypsin and amylase in intestinal contents were much higher in chickens with high bodyweight and growth rates compared with chickens with low bodyweight and growth rates [9]. Furthermore, bodyweight also has been reported to be related to the composition of the gut microbiota in humans and animals [10, 11]. The results of Ding et al. and Zhao et al. demonstrated that long-term divergent selection for abdominal fat weight and bodyweight in chickens not only changed the composition of the intestinal microbiota but also affected their functional performance by enriching the relative abundance of microorganism [12, 13]. These results indicated bodyweight as an important factor that affects intestinal morphology, digestive enzyme activity, and intestinal microbes. It is also worth mentioning that intestinal morphology and microbial composition change according to the genetic background [14, 15]. In birds, broiler chickens and ducks produced for the meat purpose always have longer, heavier, and more voluminous intestines compared with those of egg-laying fowl, such as White Leghorn chickens and wild ducks [16]. The parents also made an influence on gut microbiota of offspring since the small intestine is affected by both the maternal environment and parental diet during fetal and perinatal development in mammals [17–21].

Most of the studies that investigated the influence of parents on their offspring focused on the female parent. Recently, this focus has moved toward the role of the male parent in the phenotype of the offspring. For instance, in humans, it has been shown that a father’s body mass index (BMI) or bodyweight is independently associated with the BMI of the offspring [22]. Another study showed a stronger association in BMI between father and son than between mother and son [23]. Between two and 5 years, the height growth velocity of children was also more significantly associated with paternal height [22]. Therefore, male parent is also a critical factor participating in affecting the phenotype of offspring. Parental bodyweight is an important guarantee for the desirable traits of offspring, since many traits, such as bodyweight, growth rate, and liver weight, are related to paternal weight [24–26]. Paternal adiposity has been reported to be associated with the development of obesity and insulin resistance in offspring [27, 28]. These result suggested that paternal weight may also impact the gut function of the offspring. Therefore, it is reasonable to speculate that paternal body weight may also affect the intestinal function and inhabiting microorganisms of the offspring in meat ducks. The current study explored the influence of the paternal bodyweight on the offspring intestine by comprehensively assessing the function of the small intestine and the cecal microorganisms of offspring.

## Results

### Comparison of histological structure and digestive function of the small intestine of offspring

**Fig. 1:**
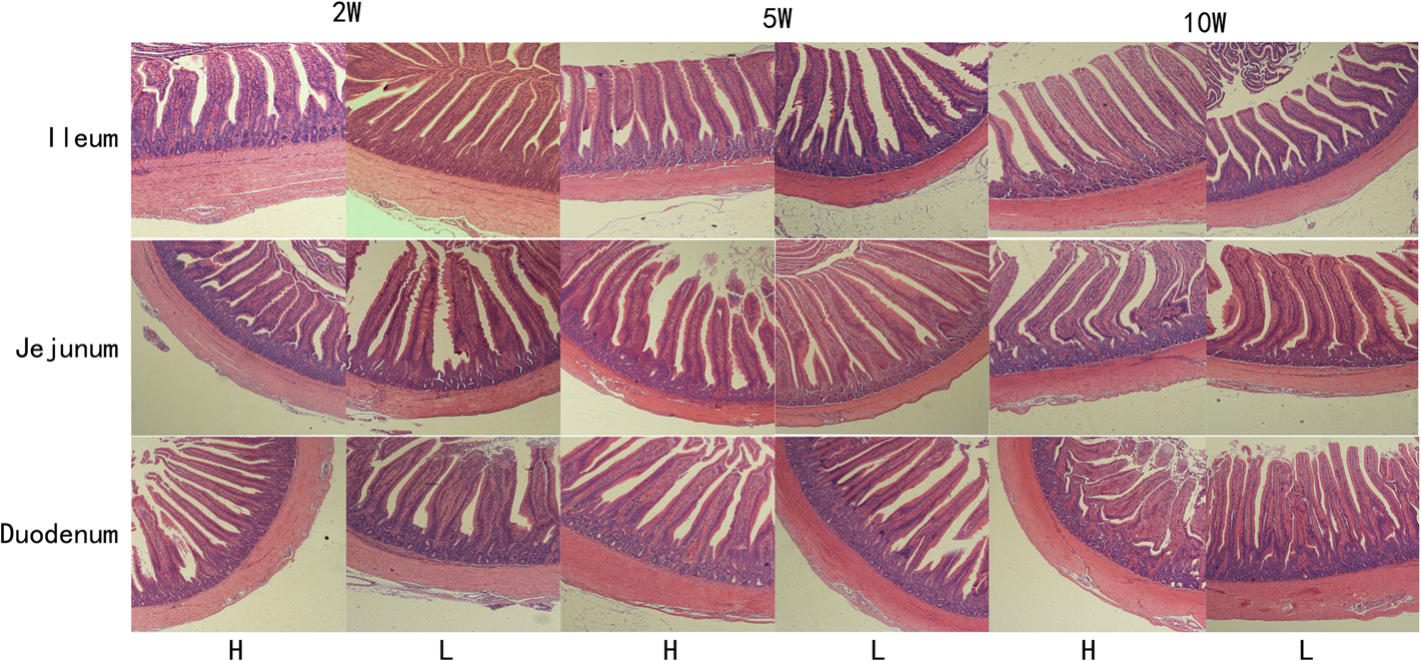
The histological observations of the small intestines of male offspring. 2 W: 2 week of age, 5 W: 5 week of age, 10 W: 10 week of age. H: High bodyweight group; L: Low bodyweight group

As shown in Table 1, both the liver (*P* < 0.01) and abdominal fat (*P* < 0.05) weights of male offspring (2 weeks of age) differed significantly between offspring of the male parent with high body weight (group H) and offspring of the male parent with low body weight (group L). However, concerning female offspring, only the body weight of group H at 5 weeks of age was significantly lower than that of group L (*P* < 0.05). These results suggested that paternal weight of ducks may exert a more pronounced effect on male than on female offspring. Therefore, male ducks were selected for the follow-up study. To achieve a better understanding of the structural differences of the small intestine, H&E staining was conducted to compare the villi and crypt of the small intestine. Morphological observations of the small intestine are illustrated in Fig. 1. The villus height (VH) and crypt depth (CD) were measured and the results are presented in Table 2. VH of jejunum of group H significantly exceeded that of group L at 10 weeks of age (*P* < 0.05). Moreover, at 2 weeks of age, the ratio of VH/CD (VC) of the duodenum in group H also significantly exceeded that of group L (*P* < 0.05).

**Table 1:** Bodyweight, liver weight, and abdominal fat weight of offspring

		Group	2 W	5 W	10 W
Male	Body weight(g)	H	433.90 ± 22.45	1893.00 ± 79.19	3130.20 ± 223.48
		L	431.24 ± 25.69	1799.60 ± 142.60	3002.00 ± 90.94
		*P* value	0.87	0.92	0.65
	Liver weight(g)	H	15.66 ± 0.80	46.74 ± 4.44	65.52 ± 14.00
		L	20.54 ± 2.24	46.72 ± 3.74	61.70 ± 15.77
		*P* value	0.01	0.59	0.17
	Abdominal fat weight(g)	H	1.30 ± 0.32	22.42 ± 3.84	51.10 ± 28.68
		L	0.76 ± 0.31	18.28 ± 4.70	32.06 ± 7.28
		*P* value	0.03	0.99	0.38
Female	Body weight(g)	H	458.26 ± 6.57	1396.60 ± 132.74	2777.80 ± 204.09
		L	437.54 ± 22.22	1766.40 ± 121.82	2720.80 ± 153.20
		*P* value	0.08	0.03	0.58
	Liver weight(g)	H	18.02 ± 2.36	49.84 ± 6.53	48.98 ± 5.56
		L	18.06 ± 1.72	49.34 ± 4.56	54.16 ± 15.02
		*P* value	0.84	0.49	0.19
	Abdominal weight(g)	H	1.34 ± 0.29	20.72 ± 5.95	59.44 ± 14.04
		L	1.30 ± 0.48	16.16 ± 4.28	49.74 ± 12.74
		*P* value	0.88	0.13	0.39

Note: H refers to group H, L refers to group L, 2 W refers to 2 weeks of age; 5 W refers to 5 weeks of age; 10 W refers to 10 weeks of age

**Table 2:** Small intestine morphology of male offspring of ducks at 2, 5, and 10 weeks of age

			Villus height (μm)	Crypt depth (μm)	Villus height/Crypt depth	Intestine thickness (μm)
2 W	Ileum	H	561.83 ± 159.73	212.13 ± 26.38	2.69 ± 0.83	260.83 ± 35.13
		L	601.61 ± 40.02	200.70 ± 16.14	3.03 ± 0.14	249.60 ± 7.27
		*P* value	0.70	0.56	0.52	0.60
	Jejunum	H	721.38 ± 96.77	149.54 ± 22.56	4.94 ± 0.37	238.45 ± 6.31
		L	630.69 ± 110.83	196.06 ± 41.35	3.51 ± 1.38	277.71 ± 63.42
		*P* value	0.35	0.16	0.16	0.30
	Duodenum	H	876.68 ± 67.99	208.04 ± 21.46	4.54 ± 0.56	311.51 ± 44.77
		L	735.46 ± 89.78	243.28 ± 15.88	3.09 ± 0.31	327.49 ± 14.66
		*P* value	0.10	0.08	0.02	0.60
5 W	Ileum	H	796.40 ± 161.72	180.98 ± 4.44	4.55 ± 0.89	260.46 ± 6.73
		L	748.70 ± 43.48	198.45 ± 19.35	3.94 ± 0.31	305.32 ± 72.39
		*P* value	0.73	0.34	0.45	0.50
	Jejunum	H	1129.52 ± 98.97	227.85 ± 18.62	5.01 ± 0.17	264.51 ± 19.84
		L	996.26 ± 204.34	212.72 ± 16.95	4.70 ± 0.62	242.92 ± 11.74
		*P* value	0.37	0.36	0.46	0.20
	Duodenum	H	1159.96 ± 76.61	220.82 ± 28.28	5.42 ± 1.07	287.45 ± 42.36
		L	1120.97 ± 127.29	269.10 ± 31.74	4.29 ± 0.68	315.50 ± 56.67
		*P* value	0.67	0.12	0.20	0.50
10 W	Ileum	H	755.88 ± 31.57	215.26 ± 52.94	3.64 ± 0.71	451.44 ± 128.98
		L	632.75 ± 125.11	148.96 ± 14.78	4.31 ± 0.44	246.14 ± 41.65
		*P* value	0.31	0.23	0.37	0.20
	Jejunum	H	1049.83 ± 5.40	211.84 ± 17.45	5.31 ± 0.57	339.10 ± 60.53
		L	828.11 ± 33.93	163.52 ± 11.23	5.30 ± 0.22	262.07 ± 22.94
		*P* value	0.01	0.08	0.99	0.20
	Duodenum	H	1023.59 ± 67.05	282.27 ± 52.07	3.72 ± 0.91	439.68 ± 73.37
		L	1150.33 ± 68.74	247.31 ± 1.61	4.67 ± 0.32	451.52 ± 72.12
		*P* value	0.20	0.44	0.30	0.90

Note: H refers to group H, L refers to group L, 2 W refers to 2 weeks of age; 5 W refers to 5 weeks of age; 10 W refers to 10 weeks of age

**Fig. 2:**
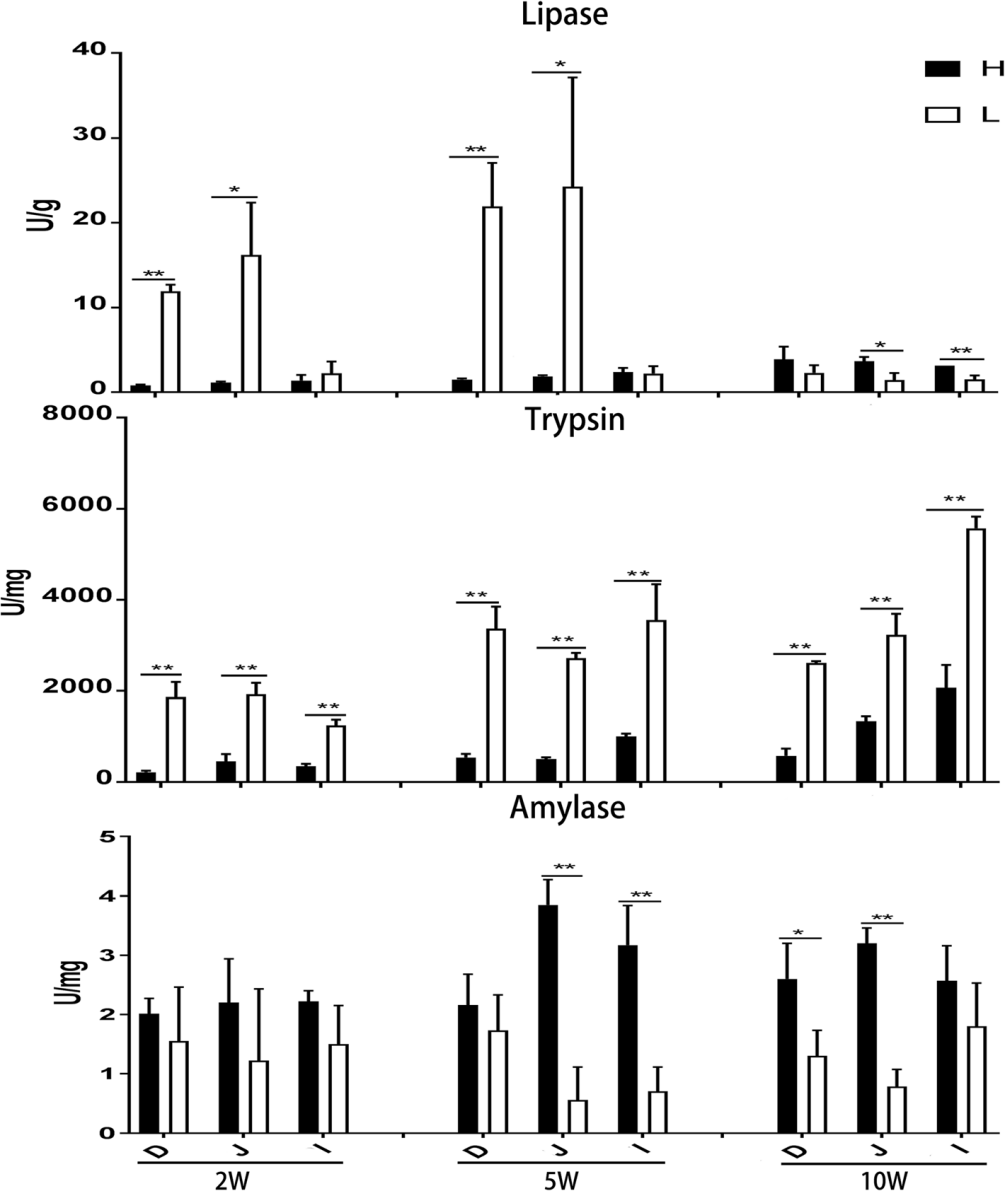
Digestive activities of the small intestine of male offspring. D: duodenum, J: jejunum, I: ileum, * *p*-value < 0.05, ** *p*-value < 0.01. All other definitions can be found on the legend of Fig. 1

To further understand differences of the digestive function of the small intestine, the digestive enzyme activities in the jejunum, ileum, and duodenum were measured. As shown in Fig. 2, compared with ducks in group H, the activities of lipase of jejunum were much higher in ducks of 2 and 5 weeks of age in group L; however, this effect was reversed in 10 weeks old ducks (Fig. 2a). Moreover, the activities of small intestinal trypsin of ducks in group L were also significantly higher than that of ducks in group H at three different ages (Fig. 2b). In contrast, amylase activities in the jejunum were significantly higher in 5 and 10 week ducks of group H compared with ducks of group L (Fig. 2c).

### Comparison of the microbial complexity

A total of 1,692,094 clean tags were obtained from 30 samples after filtering for quality. These clean tags were clustered into 193 to 478 operational taxonomic units (OTUs) for each sample at a 97% sequence similarity threshold. Microbial complexity in the cecum was estimated based on alpha-diversity indices (Chao1, Ace, Shannon, and Simpson). As shown in Table 3, at an age of 2 weeks, the ACE, Chao1, and Shannon indexes of group H were slightly lower than those of group L; however, they were higher than those of group L at the ages of 5 weeks and 10 weeks, the Simpson index showed the opposite result. This indicates that the richness of cecum microbiota in group H was first lower (2 weeks) and then higher (5 and 10 weeks) than that of group L; however, these differences between groups were as not significant. Moreover, all of these diversity indices increased significantly at 5 weeks compared with 2 weeks(*p* < 0.05) but remained stable between 5 and 10 weeks of age.

**Table 3:** Alpha-diversity indices

		2w	5w	10w
OTU	H	222.00 ± 22.18	391.00 ± 7.52	387.20 ± 32.94
	L	215.80 ± 16.35	377.00 ± 19.40	375.20 ± 31.46
	*P* value	0.63	0.17	0.57
ACE	H	242.11 ± 15.30	402.61 ± 9.74	408.17 ± 26.22
	L	244.01 ± 18.45	393.62 ± 21.38	402.44 ± 24.33
	*P* value	0.86	0.42	0.73
Chao1	H	247.07 ± 13.43	404.74 ± 12.76	415.14 ± 24.46
	L	252.81 ± 13.96	402.62 ± 22.70	407.77 ± 21.69
	*P* value	0.53	0.86	0.63
Simpson	H	0.14 ± 0.07	0.05 ± 0.02	0.04 ± 0.01
	L	0.11 ± 0.04	0.06 ± 0.01	0.07 ± 0.07
	*P* value	0.39	0.44	0.39
Shannon	H	2.96 ± 0.33	4.08 ± 0.23	4.28 ± 0.27
	L	3.01 ± 0.25	3.86 ± 0.24	4.10 ± 0.49
	*P* value	0.81	0.81	0.50

Note: H refers to group H, L refers to group L, 2 W refers to 2 weeks of age; 5 W refers to 5 weeks of age; 10 W refers to 10 weeks of age

### Comparison of the microbial community composition

Rarefaction curves were constructed to verify whether the microbiota of the 30 samples was large enough to estimate phenotype richness. This result showed that the rarefaction curves tended to attain the saturation plateau, suggesting that the microbiota of the 30 samples was sufficiently large to estimate the phenotype richness and microbial community diversity (see Additional file 1).

All sequences were classified into 13 phyla, three of which were most frequent (relative abundance > 1%): Firmicutes, Bacteroidetes, and Proteobacteria. Except for ducks of group H at 2 weeks of age when Bacteroidetes was the dominant phylum, the cecal microbial population of ducks was dominated by phylum Firmicutes in weeks 2, 5, and 10 in both group H and group L, making up an average of 47% of the microbial population. Furthermore, the ratio of Firmicutes/Bacteroidetes in group H was significantly higher than in group L (2 weeks), while at 10 weeks of age, it was higher in group L (*P* < 0.05). Compared with group L, the relative abundance of Proteobacteria was significantly lower in group H (2 weeks), and at 10 weeks of age, it was significantly higher in group H (*P* < 0.05) (see Additional file 2).

**Fig. 3:**
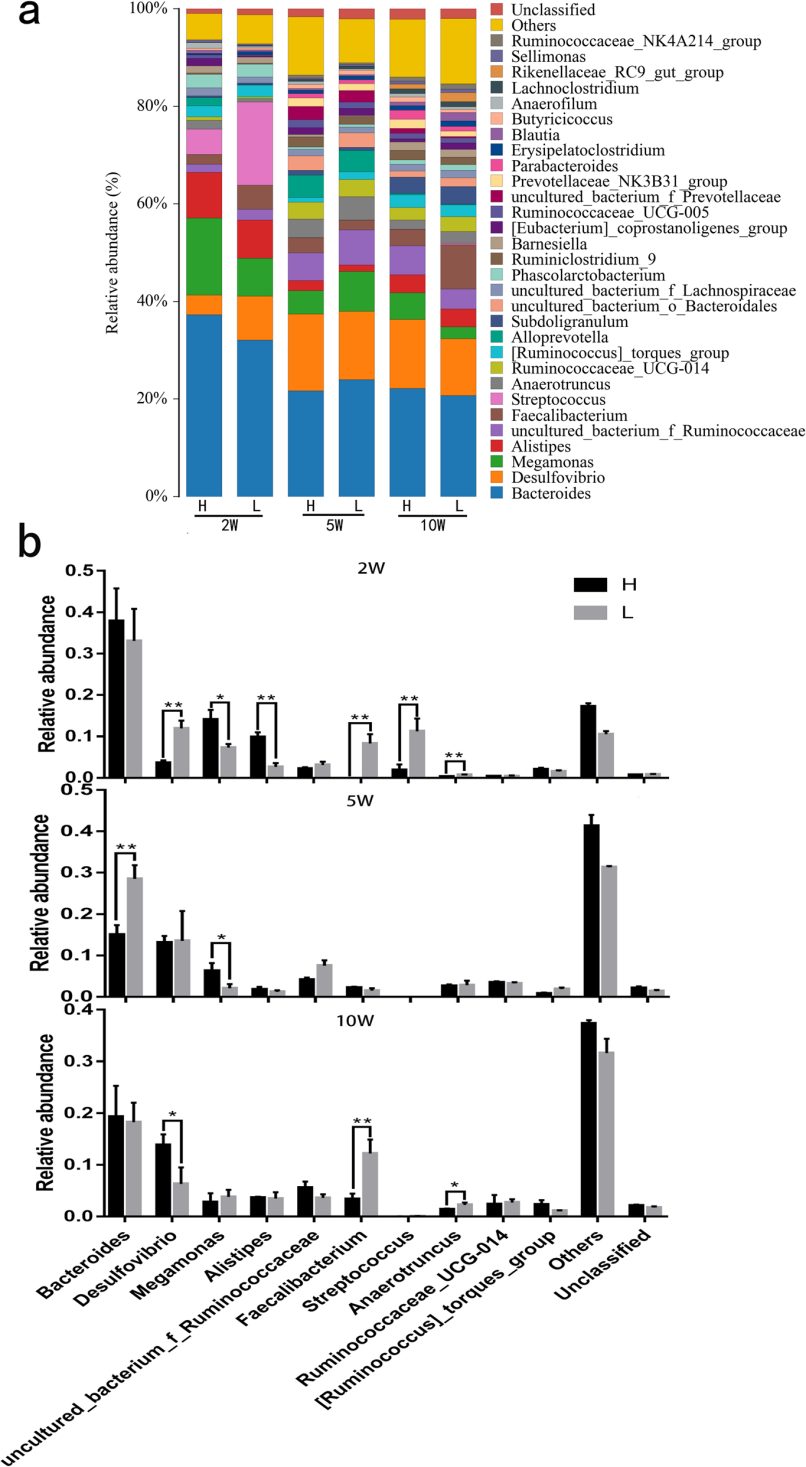
Bacterial community composition and differences of abundance at the genus level. **a** Bacterial community composition at the genus level of different samples. These are the top 30 genera with the highest abundance and the rest of them were combined into “other”; **b** Changes of microbial genera of the cecum at 2, 5, and 10 weeks of age. All definitions can be found on the legend of Fig. 1

At the genus level, *Bacteroides* was the dominant genus in the cecum of male ducks at 2, 5, and 10 weeks of age and the number of the genus with abundance above 1% increased with age (Fig. 3a). At 2 weeks of age, the abundance of *Streptococcus, Desulfovibrio, Faecalibacterium*, and *Anaerotruncus* in group H was significantly lower than in group L, while *Megamonas* and *Alistipes* in group H were significantly higher than in group L (*P* < 0.05). At 5 weeks of age, the relative abundance of *Bacteroides* in group H was significantly lower than in group L, and the relative abundance of *Megamonas* was significantly higher in group H than in group L (*P* < 0.05). At 10 weeks of age, compared with group L, the relative abundance of *Desulfovibrio* was significantly higher in group H, while *Faecalibacterium* and *Anaerotruncus* were significantly lower in group H (*P* < 0.05) (Fig. 3b).

Microorganisms can adapt and modify their surroundings via their metabolic products. In this study, reconstruction via unobserved states (PICRUSt) software was used to infer the functional genome composition of samples, and then, the metabolism of the microorganisms could be assumed in reference to KEGG databases according to the composition of genome function. The results showed that the most abundant pathways in groups H and L at 2, 5, and 10 weeks of age were carbohydrate metabolism, global and overview maps, and amino acid metabolism (see Additional file 3).

**Fig. 4:**
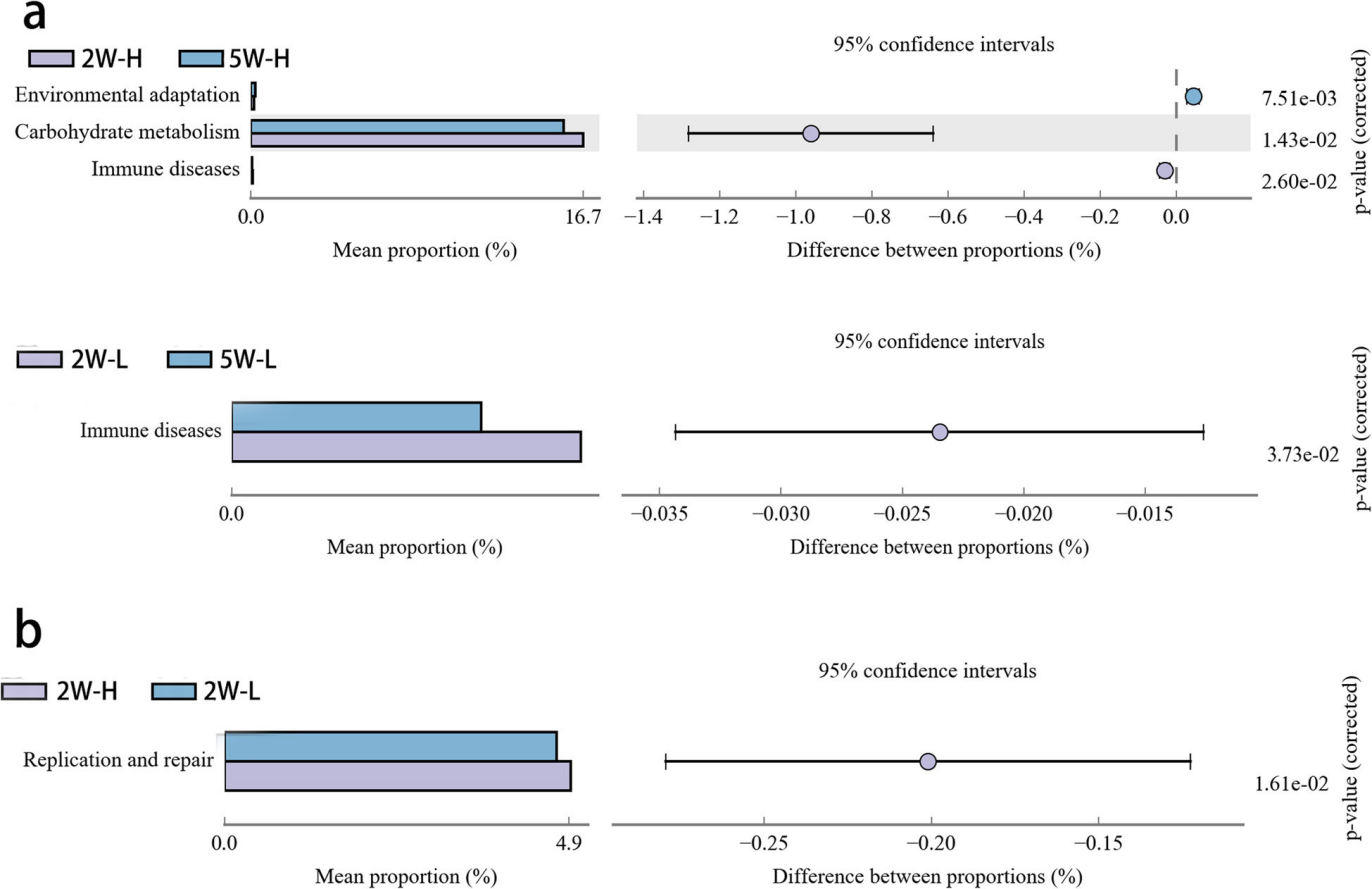
KEGG pathway comparison. Error bars represent the abundance of microorganisms and different colors represent different groups. 2 W-H: High bodyweight group at 2 week of age, 2 W-L: Low bodyweight group at 2 week of age, 5 W-H: High bodyweight group at 5 week of age, 5 W-L: Low bodyweight group at 5 week of age. **a** Pathways that changed with age. **b** Pathways with different proportions between groups

Compared with 2 weeks, microorganisms enriched in environmental adaptation pathways in group H increased sharply at 5 weeks of age, while both carbohydrate metabolism and immune disease-related microorganisms decreased significantly. However, in group L, only microorganisms related to immune diseases decreased significantly at 5 weeks of age (Fig. 4a). Between 5 and 10 weeks, none of the pathways with a significant difference in the number of enriched microorganisms. At the age of 2 weeks, the number of microorganisms in the replication and repair pathway in group H was significantly higher than that of group L (Fig. 4b). However, no significant difference between group H and group L was found in the enriched pathway at 5 and 10 weeks of age.

### Correlation analysis

**Fig. 5:**
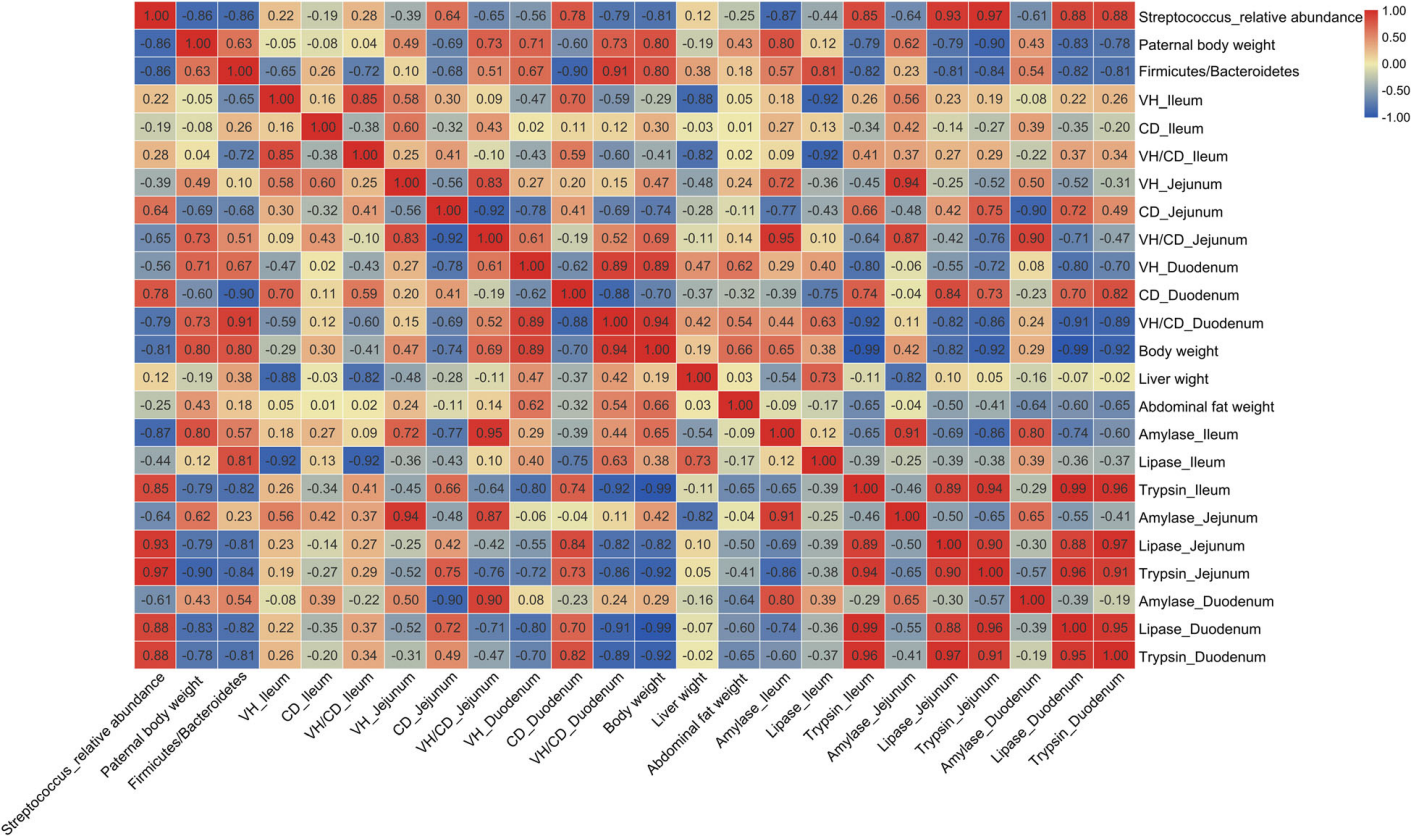
Correlation analysis between microbiota and other biochemical parameters. VH_Ileum: Villus height of the ileum; CD_Ileum: Crypt depth of the ileum; VH/CD_Ileum: ratio of villus height to crypt depth of the ileum. VH_Jejunum: Villus height of jejunum; CD_Jejunum: Crypt depth of jejunum; VH/CD_Jejunum: ratio of villus height to crypt depth of the jejunum. VH_Duodenum: Villus height of the duodenum; CD_Duodenum: Crypt depth of the duodenum; VH/CD_ Duodenum: ratio of villus height to crypt depth of the duodenum

The results of microbial composition, abdominal fat weight, intestinal morphology, and digestive enzyme activity measurement showed that the bodyweight of the male parent mainly affected the intestinal function of 2-week-old male offspring. Therefore, a correlation analysis was conducted between microbiota and other biochemical parameters at 2 weeks of age. As shown in Fig. 5, the paternal body weight was significantly and negatively correlated with the relative abundance of *Streptococcus* and the activities of trypsin in the jejunum and lipase in the duodenum. Moreover, the activities of lipase and trypsin in the jejunum and duodenum were significantly and negatively correlated with the proportion of Firmicutes/Bacteroidetes and the relative abundance of *Megamonas* and *Alistipes*. However, it was positively related to the relative abundances of *Desulfovibrio*, *Faecalibacterium*, *Streptococcus*, and *Anaerotruncus*. There was also a significant correlation among genera with significant differences in relative abundance between groups H and L.

## Discussion

In the poultry industry, good intestinal health is of great importance to achieve both target growth rates and feed efficiency [2]. The experiment conducted in the present study investigated the effect of parental bodyweight on the intestinal function of male offspring in ducks. The result of morphological analysis of the small intestine showed that the VH of the jejunum in group H exceeded that of group L, and the difference was significant at 10 weeks of age. Villi and crypts are structural units of the small intestine, and the actual surface area available for both the digestion and absorption of nutrients is determined by villi [29]. Villous atrophy indicates that the villus absorptive cells decrease and the secretory cells increase, which causes a deterioration of absorptive capacity [30, 31]. Thus, the higher VH of the jejunum indicates a higher absorptive capacity and this result suggests that the increase of paternal bodyweight may result in an improvement of digestive capacity by improving the VH of male offspring.

Digestion of the feed in the intestine is achieved by pancreatic proteases, peptidases, lipase, and amylase contributes further to the breakdown of nutrients [32]. Nitsan et al. found that compared with low bodyweight chickens, high bodyweight chicken showed much higher trypsin and amylase activities in the intestinal contents 3 days after hatching [9]. The result of the present study showed that the ducks of group L had higher trypsin activity, while the ducks of group H had higher amylase activity. The digestive ability of the small intestine in response to changes in physiological needs has been demonstrated in both mammals and birds, and has been mainly linked to nutrient absorption and organ structure [33]. Therefore, the differences of digestive enzyme activities between group H and group L indicate the existence of differences in nutritional requirements between ducks of both groups. Lipase, trypsin, and amylase are related to the digestion of different nutrients. Amylases are a group of enzymes that are responsible for the breakdown of starches into sugars [34]. Lipases are serine hydrolases and are defined as triacylglycerol acylhydrolases that participate in the hydrolysis of fat [35]. Furthermore, the processes of protein digestion in the small intestine is mainly affected by trypsin [36]. The results of the present study showed that the lipase and trypsin activities of ducks in group H were lower while the amylase activities were higher than in ducks of group L. This indicated that ducks in group H had a higher starch digestion ability, but that their digestion ability of fat and protein was weaker than ducks of group L.

Both the diversity and function of microorganisms in the gastrointestinal tract is subject to ongoing research, and it has been recognized to play a role in nutrition absorption, intestinal development, and host physiological regulation [37]. The ceca consist of two blind pouches that make it difficult for food to pass through; therefore, it is an ideal habitat for a diverse microbiome [38]. Consequently, the cecum contents were collected to analyze the microbial composition. This study presents the first inventory of intestinal microbiota in the Nonghua sheldrake meat duck. Based on this study, except for group H at 2 weeks when Bacteroidetes was the dominant phylum, the microbial population of ducks was dominated by the phylum Firmicutes during weeks 2, 5, and 10 in both groups H and L, and it supplied an average of 47% of the microbial population, which differed from the Peking Duck in which Firmicutes accounts for an average of 96% of the microbial population [39]. However, VasaΪ et al. identified phylum Bacteroidetes as the dominant phylum of both Muscovy and Pekin ducks [40]. The composition and activity of the intestinal microbiome are easily affected by many factors and therefore, these differences may be the result of breed, environment, and diet. The composition of intestinal microorganisms becomes increasingly complex with increasing host age, and intestinal microorganisms of different species follow different rules of variation [39, 41]. In chicken, at hatching (day 1), no bacteria were detected in any part of the gastrointestinal tract, but by day 3, a large number of *Streptococcus faecalis* and *Escherichia coli* could be isolated from all parts of their gastrointestinal tract. Microbial communities were established in the small intestine within about 2 weeks. In contrast, cecum microbial communities are only established 6–7 weeks later than in the small intestine [42]. In the present study, from weeks 5 to 10, the number of changed microorganisms decreased sharply compared with the number from 2 to 5 weeks and the microbial complexity also no longer increased. This indicated that the microbial community in the cecum of Nonghua Sheldrake ducks may be stable at around 5 weeks of age. However, a more robust experiment is required to confirm this finding.

Previous research suggested strong interaction between intestinal microorganisms and host body weight. Angelakis et al. found that the increase of *Lactobacillus* species in the gut flora of newborn broiler chicks and ducks promotes weight gain [43]. Bäckhed et al. investigated the proportion of Firmicutes and Bacteroidetes in obese mice and normal-weight mice, and found that in obese mice, the proportion of Bacteroidetes was significantly decreased (20%) while the number of bacteria in mice with normal weight was as high as 40% [44]. In the present study, compared with ducks of group L (2 weeks), the proportion of Firmicutes to Bacteroidetes was significantly higher in ducks of group H, which was reversed at 10 weeks of age. In the human gut microbiota, the increase of Firmicutes is associated with better energy absorption from the diet, while an increase in Bacteroidetes is associated with decreased energy absorption [45]. This result suggests that at the age of 2 weeks, ducks of group H have a higher absorption capacity of nutrients, and with increasing age, the absorptive capacity of ducks in group L was significantly increased and exceeded that of group H at 10 weeks of age. Also at 2 weeks, the abundance of *Streptococcus* observed in group H was significantly lower than that observed in group L. *Streptococcus* have been described as commensal bacteria in both humans and other animals. Their presence increased when bodyweight increased in response to overfeeding in Pekin and Muscovy ducks [40]. However, in cows, Fernando et al. reported an increase of *Streptococcus spp* in response to high-grain diets, especially *Streptococcus bovis* [46]. However, in the present study, the reduced abundance of *Streptococcus* in group H did not lead to weight loss. This suggests that *Streptococcus* in male ducks does not influence bodyweight, but rather affects other aspects as they have also been found to cause severe infections [47].

It has been reported that the realization of intestinal function is affected by many factors, including the small intestine structure [30, 48], digestive enzymes [13, 48], and gut microbiota [49, 50]. The analyses of intestinal structure, digestive enzyme activity, and cecum microbiological showed that the paternal bodyweight may affect the internal function of male offspring in ducks. Furthermore, the results of correlation analysis indicated that the paternal bodyweight was significantly and negatively correlated with the relative abundance of the genus *Streptococcus* and the activities of trypsin in the jejunum and lipase in the duodenum at 2 weeks of age. This result further supports the hypothesis that paternal bodyweight may affect offspring intestinal digestive enzyme activity and microbial composition.

## Conclusions

In conclusion, this study demonstrates that the significant differences in paternal bodyweight may lead to differences in the ratio of VH to crypt depth of the duodenum (2 weeks) and the villus height of the jejunum, as well as amylase activity in the small intestine at 2, 5, and 10-week-old. With regard to the microorganisms of the cecum, the relative abundance of five genera in the cecum and the number of microorganisms enriched in replication and repair pathway at 2 weeks of age were also significantly different between group H and group L. All these results preliminarily indicate that the difference of paternal body weight may affect the small intestinal function and cecal microorganism of offspring, which lays a foundation for further research on the relationship between male parent and intestinal function.

## Methods

### Animal preparation and sample collection

Parent Nonghua Sheldrake ducks were obtained from the poultry breeding farm of Sichuan Agricultural University and were divided into high bodyweight group (group H) and low bodyweight group (group L) according to the bodyweight of male ducks (*P* < 0.05; Table 4). Then, the offspring of both groups were obtained by artificial insemination. The ducklings of groups H and L were divided and reared according to sex, which means that males and females from both groups were divided into two groups (male group and female group) and online fed separately. Furthermore, all experimental ducks had free access to food and water and were subjected to the same routine immunization procedures. A commercial duckling diet was supplied ad libitum for 0–3 week-days, and after that, the normal growth feed for meat ducks was supplied. The slaughtering of five male ducks, five female ducks, from H and L groups each was performed after weighting at 2, 5 and 10 weeks, and all of these ducks were euthanized by cervical dislocation. After dissection, liver and abdominal fat were separated and immediately weighted (Table 1). At the same time, luminal contents of the duodenum, jejunum, and ileum were removed and cecal contents were collected through quick freezing by liquid nitrogen. The remaining duodenum, jejunum, and ileum were fixed with 4% polyformaldehyde solution for histological investigations.

**Table 4:** Parent bodyweight, liver weight, and abdominal fat weight

	Group	Male	Female
Body weight(g)	H	4006.67 ± 297.04	2197.14 ± 52.82
	L	3450.00 ± 80.00	2217.14 ± 52.82
	*P* value	0.04	0.78
Liver weight(g)	H	130.60 ± 86.62	62.37 ± 18.19
	L	78.00 ± 54.90	77.49 ± 27.84
	*P* value	0.43	0.43
Abdominal fat weight(g)	H	57.37 ± 44.24	15.40 ± 6.01
	L	43.17 ± 9.15	15.74 ± 6.21
	*P* value	0.62	0.48

Note: H refers to group H, L refers to group L, 2 W refers to 2 weeks of age; 5 W refers to 5 weeks of age; 10 W refers to 10 weeks of age

### Histomorphological observation

The samples fixed with 4% polyformaldehyde solution for histological comparison were washed under running water, dehydrated with an ethyl alcohol series, cleared in xylene, and embedded in paraffin wax. A Leica RM2016 microtome (Shanghai Leica Instrument Company, China) was utilized to cut specimens into slices of 4-μm thickness. Cross-sections were stained with H&E and photographed with an E100 biomicroscope (Nikon Eclipse. Japan). The small intestinal VH and CD were measured using Image Pro-Plus software (version6.0) at 40× magnification.

### Digestive enzyme assays

Samples of small intestinal digesta (duodenum, jejunum, and ileum) were homogenized (1:4 wt/vol) with 0.86% ice-cold physiological saline. Then, the homogenate was centrifuged at 2500 r/min, at 4 °C for 15 min, and the supernatant was collected and kept at 4 °C (Lipase and total protein) or − 20 °C (trypsin) until use. After the total protein concentration of the sample had been determined by the total protein assay kit of the Nanjing Jiancheng Bioengineering Institute (Nanjing, Jiangsu, China). Activities of lipase, amylase, and trypsin were determined using a corresponding diagnostic kit of Nanjing Jiancheng Bioengineering Institute (Nanjing, Jiangsu, China) and Beijing Solarbio Science & Technology according to the instructions of the manufacturers.

The trypsin activity was measured as follows: 0.015 mL of the test or blank sample supernatant, and 1.5 mL of trypsin substrate were preheated at 37 °C for 5 min, and 0.015 mL of sample homogenate medium were mixed. Then, optical density (OD) values of the 30 s and 20.5 min were measured at 253 nm, and then, the trypsin activity was calculated based on absorbance value and total protein concentration.

Lipase (EC 3.1.1.3) activity was assayed with the lipase assay kit of the Nanjing Jiancheng Bioengineering Institute. Briefly, 4 μL of sample was mixed with 200 μL of co-lipase diluted in Good’s buffer (pH = 8.0), and then incubated at 37 °C for 3–5 min. Finally, the mixture was mixed with 50 μL of chromogenic substrate diluted in tartaric acid buffer (pH = 4.0) and incubated at 7 °C for 2 min. The change in absorbance of each well was continuously monitored for 2 min at the measured wavelength.

Protease activity was analyzed using the method described below: After mixing 100 μL sample or ultrapure water with 1.5 mL substrate buffer, at 37 °C in a water bath for 7.5 min. Then, 500 μL iodine application solution and 3 mL ultrapure water were added and mixed. After that, the OD value at 660 nm was measured.

### DNA extraction, 16S rRNA gene amplification and high-throughput sequencing

Power Soil DNA Isolation Kit (MO BIO Laboratories) was used to extract the total bacterial DNA of samples according to the manufacturer’s instruction. The ratios of 260 nm / 280 nm and 260 nm / 230 nm were used as an indicator of both DNA quality and quantity. After the quality evaluation of the extracted DNA, until further processing, the extracted DNA was stored at − 80 °C.

The the bacterial 16S rRNA gene’ V3-V4 region was amplified by using the universal primer (forward primer, 5′-ACTCCTACGGGAGGCAGCA-3′; reverse primer, 5′-GGACTACHVGGGTWTCTAAT-3′) to combine the adapter sequences and barcode sequences. The total volume of PCR amplification was 50 μL, including 10 μL buffer, 10 μL high GC enhancer, 10 μM of each primer, 0.2 μL Q5 high-fidelity DNA polymerase, 1 μL dNTP, and 60 ng genomic DNA. The thermal cycling conditions were as followed: firstly, denaturation at 95 °C for 5 min, followed by reaction at 95 °C for 1 min for 15 times, and then 50 °C for 1 min, 72 °C for 1 min, finally extension at 72 °C for 7 min. VAHTSTM DNA clean beads was used to purify the first-step PCR products. A second-round PCR was then conducted, the amplification system is as follows: 8 μL ddH_2_O, 20 μL 2× Phμsion HF MM, 10 μM of each primer, and 10 μL PCR products from the first-step PCR. The thermal cycling conditions were as follows: 98 °C for 30 s, at 98 °C for 10 s for 10 cycles, and then 65 °C for 30 s min, 72 °C for 30 s, finally extension at 72 °C for 5 min. After the second PCR amplification, Quant-iT™ dsDNA HS reagent was used to quantified the PCR products and the PCR products were pooled.

Finally, PCR products pool was sequenced using the Illumina Hiseq 2500 platform (2 × 250 paired ends).

### Bioinformatics analysis

FLASH (version 1.2.11) was used to assemble PE reads; only sequences that overlapped for more than 10 bp were assembled according to their overlapping sequence. Sequences with an overlap default ratio exceeding 20% in the overlap region were discarded. Then, Trimmomatic (version 0.33) was used to quality filter the raw fastq files. Finally, the chimaeras were removed via UCHIME (version 8.1) to obtain high-quality Tags sequences.

Sequence clustering at the 97% similarity level (USEARCH, version 10.0) used 0.005% of all sequences as a threshold to filter the OTUs. The reads from filtered OTUs were processed using QIIME (version1.8) to construct a representative sequence for each OTU. The defined OTUs were assigned to different taxonomic levels (i.e., phylum and genus) at a cut off of 97% compared with the SILVA and UNITE databases. Moreover, the clustered OTUs were also used to construct rarefaction curves and the Shannon and Simpson diversity indices, abundance-based coverage estimators (ACE), Chao 1 richness (Mothur, version v.1.30) were calculated.

### Statistical analysis

Changes in bacterial abundance were analyzed by repeated-measures ANOVA analysis accompanied by Tukey’s honestly significant difference posthoc test. Furthermore, differences of digestive enzyme activity, VH, crypt depth, bodyweight, liver weight, and abdominal fat weight were analyzed by ANOVA or nonparametric test according to their test results of homogeneity of variance. IBM SPSS Statistic (version 20) was used for all statistical analyses. Pearson correlation coefficients of intestinal microflora with other VH, crypt depth, VH/CD, bodyweight, liver weight, abdominal fat weight, and digestive enzyme activity were also determined by IBM SPSS Statistic (version 20). The differences were considered to be significant at *P* < 0.05. Results are presented as means ± standard deviation (S.D.).

## Supplementary information

**Additional file 1: Figure S1.** Rarefaction curves for all samples

**Additional file 2: Figure S2.** The bacterial community composition and abundance differences at phylum levels

**Additional file 3: Figure S3.** KEGG classification histogram of all group

## Data Availability

All data generated or analysed during this study are included in this published article [and its supplementary information files].

## Abbreviations

Group H: Offspring of male parents with high-body weight
group L: Offspring of male parents with low-body weight
VH: Villus height
CD: Crypt depth
VC: Villus height/ crypt depth
2 W: 2 Weeks
5 W: 5 Weeks
10 W: 10 Weeks
OUT: Operational taxonomic unit
ACE: Abundance-based coverage estimators
OD: Optical density
