# Supplementary figures and images for: Paternal weight of ducks may have an influence on offspring’ small intestinal function and cecal microorganisms
Source: BMC Microbiol. 2020 Jun 5;20:145. doi: 10.1186/s12866-020-01828-1 (PMC7275315; doi:10.1186/s12866-020-01828-1)

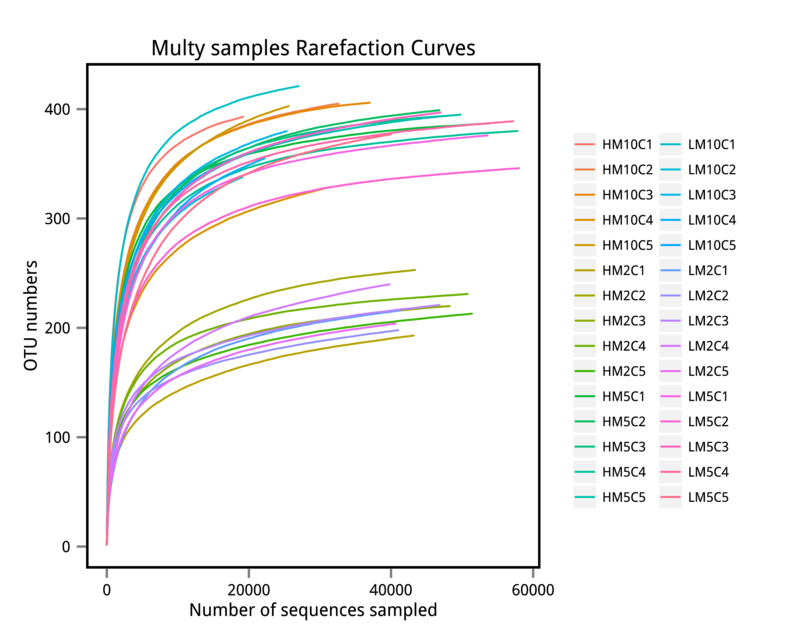


Figure S1. Rarefaction curves for all samples

Supplement: Supplementary file 1 — Additional file 1: Figure S1. Rarefaction curves for all samples [file 12866_2020_1828_MOESM1_ESM.docx]

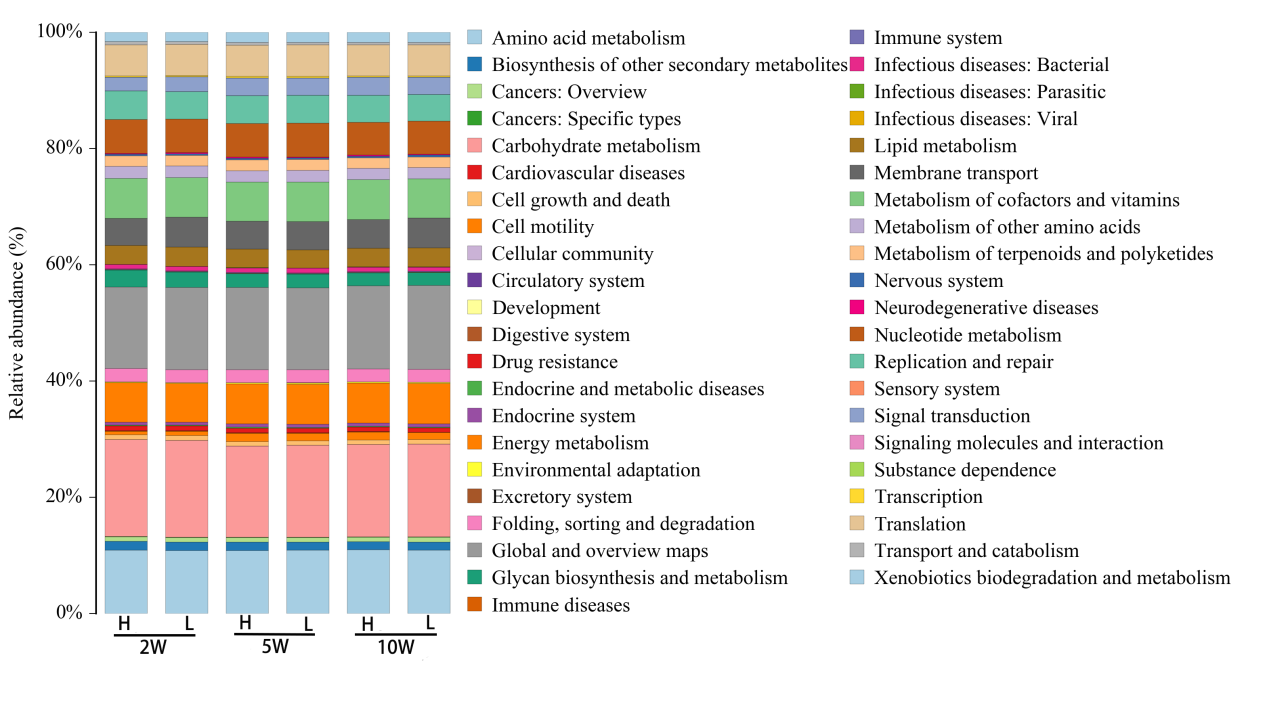


Figure S3. KEGG classification histogram of all samples.

Supplement: Supplementary file 3 — Additional file 3: Figure S3. KEGG classification histogram of all group [file 12866_2020_1828_MOESM3_ESM.docx]
