# Supplementary material for: Paternal weight of ducks may have an influence on offspring’ small intestinal function and cecal microorganisms
Source: BMC Microbiol. 2020 Jun 5;20:145. doi: 10.1186/s12866-020-01828-1 (PMC7275315; doi:10.1186/s12866-020-01828-1)
Supplement: Supplementary file 2 — Additional file 2: Figure S2. The bacterial community composition and abundance differences at phylum levels [file 12866_2020_1828_MOESM2_ESM.docx]

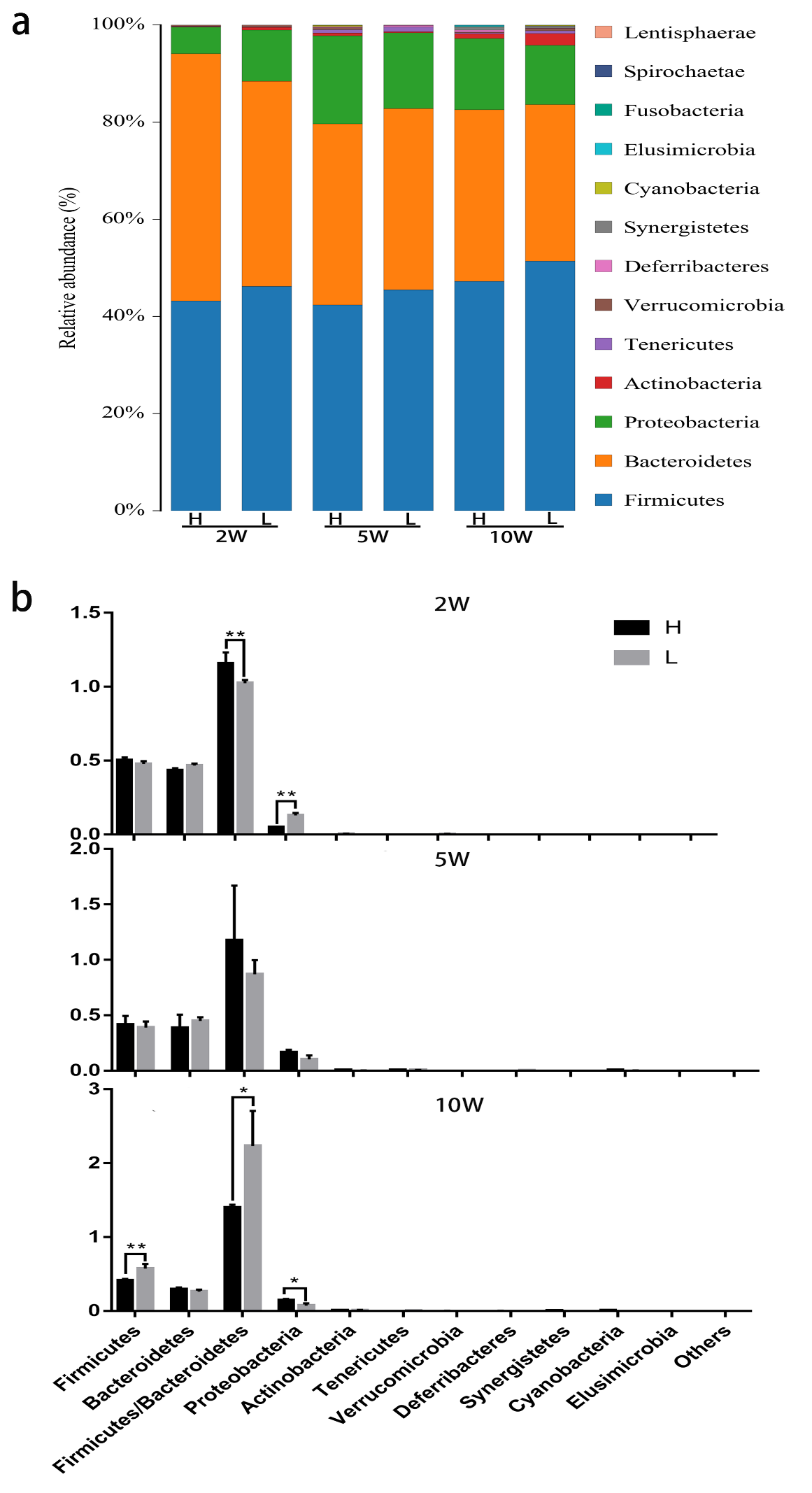


Figure S2: The bacterial community composition and abundance differences at phylum levels. a: The bacterial community composition at phylum levels of different samples; b: Relative abundance of microorganisms at phyla level.
